# Wrangling Reactive Nitrogen: Strategies for Mitigating Pollution

**DOI:** 10.1289/ehp.120-a200

**Published:** 2012-05-01

**Authors:** Tim Lougheed

**Affiliations:** **Tim Lougheed** has worked as a freelance writer in Ottawa, Canada, since 1991. A past president of the Canadian Science Writers’ Association, he covers a broad range of topics in science, technology, medicine, and education.


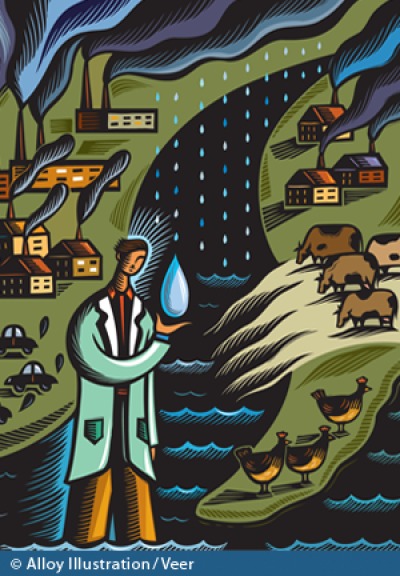
Central California’s agricultural bounty, which has stocked grocery shelves across North America for decades, has also stocked the region’s groundwater supply with nitrates, according to a two-year study by researchers at the University of California, Davis.^^1^^ Aquifers that provide drinking water for thousands of people regularly exceed the state’s nitrate concentration safety limits,^^1^^ potentially raising the risk of thyroid problems, adverse birth outcomes, circulatory problems, and cancer.^^2^^

The study goes by the name SBX2 1, after the 2008 California Senate bill^^3^^ that called for the California State Water Resources Control Board to deliver a report on nitrate pollution in drinking water—an issue flagged by the state government some 20 years earlier.^^4^^ Prepared by the UC Davis Center for Watershed Sciences, the report draws data from some two dozen private and government agencies, assembling a database of 100,000 samples from nearly 20,000 wells. The results were formally announced at a 13 March 2012 workshop.

The report concludes that regulatory actions to date have failed to contain nitrate pollution of groundwater, which could in fact grow worse in coming decades. Nevertheless, the authors add, there do exist low- and moderate-cost options that could yet effectively control pollution—provided the appropriate political and industrial will is brought to bear to implement them. “I’m very optimistic that in another twenty years there won’t be another report to the legislature,” concluded principal investigator Thomas Harter after the three-hour workshop, which included a panel discussion to lay out a wide range of approaches to the problem.

This sentiment was reflected in the Winter 2012 edition of *Issues in Ecology*, which was subtitled “Excess Nitrogen in the U.S. Environment: Trends, Risks, and Solutions.”^^5^^ A team of authors led by Eric A. Davidson, executive director and senior scientist of The Woods Hole Research Center, reported there have been “important successes in reducing nitrogen emissions to the atmosphere, and this has improved air quality.” They also noted that effective options have been identified for reducing nitrogen losses from agriculture, “although political and economic impediments to their adoption remain.”

## What Is Reactive Nitrogen Pollution?

The effects of nitrogen pollution can be found everywhere that nitrogen has transformed the air, water, or land. The culprit in each case is reactive nitrogen, meaning any form of the element other than the nonreactive atmospheric gas N_2_. Where reactions occur, the most common products are nitrous oxide (N_2_O), nitrite (NO_2_), nitrate (NO_3_), ammonia (NH_3_), and nitrogen oxides (NO_X_).

The largest proportion of reactive nitrogen in the environment comes from agriculture.^^6^^ In 1909 the Nobel prize–winning German chemists Fritz Haber and Carl Bosch developed the Haber process, which enabled the industrial-scale production of ammonia for use in fertilizer and explosives. Over the next century the price of ammonia plummeted, and the world now uses upward of 400 billion pounds of the stuff every year, most of it winding up on farmer’s fields.^^7^^ Other anthropogenic sources of reactive nitrogen include industry, transportation, and electricity generation; natural inputs include lightning and bacterial nitrogen fixation.^^5^^

A 2007 review by the United Nations Environment Programme and The Woods Hole Research Center showcased the difficulties caused by reactive nitrogen in different settings around the world.^^8^^ Wherever internal combustion engines flourish, for example, they emit large volumes of NO_X_, which has been linked with respiratory disease, diminished heart and lung function, and reproductive problems.^^2^^^^,^^^^9^^ Nitrogen compounds also react with other air pollutants to form toxic ozone and particulate matter.^^8^^

In the developed world, legislative measures requiring manufacturers to limit these emissions have met with noteworthy success. According to *Reactive Nitrogen in the United States: An Analysis of Inputs, Flows, Consequences, and Management Options*, a 2011 report by the U.S. Environmental Protection Agency (EPA) Science Advisory Board, agency air-quality tests conducted over the past decade showed that between 1990 and 2002, emissions of NO_X_ associated with fossil fuel burning dropped by one-third, with emissions associated with electric power generation dropping by 70%.^^10^^

Similarly, researchers in Nebraska’s Platte River Valley have spent the last few years demonstrating the virtue of one of the key recommendations in the SBX2 1 report. By converting about 17% of the study area from furrow irrigation to better-controlled sprinkler irrigation, less fertilizer was necessary and crop yields rose, which meant plants took up more nitrogen from that area. Consequently, groundwater nitrate concentrations decreased slowly but steadily between 1994 and 2003, leading the researchers to predict that in several decades it will be well below any hazardous level.^^11^^

Elsewhere, however, communities are still struggling to rein in reactive nitrogen. Residents of the heavily populated Chesapeake Bay watershed must cope with nitrogen sources such as power-plant smokestacks, wastewater treatment plants, and farm runoff. Although the quality of the area’s air and water has improved since the 1990s, ongoing development and population growth have made it difficult to lower the amount of nitrogen entering the bay, where elevated acidity and nitrate levels have damaged local fisheries. Six states plus the District of Columbia abut this huge estuary, complicating regulatory efforts to reduce these inputs.^^8^^

Meanwhile, the U.S. Department of Agriculture’s Conservation Effects Assessment Program has obtained conflicting results on the best way to lower nitrate concentrations in the Mississippi River basin, which are ultimately responsible for the massive hypoxic region—dubbed a “dead zone”—in the Gulf of Mexico.^^12^^ Attempts to limit nitrogen runoff in the upper river basin have met with limited success, with nitrate concentrations at some sites along the river increasing by as much as 76% since 1980. However, major tributaries draining into the river have shown no such increase, and concentrations in the Mississippi itself were found to increase seasonally after spring snowmelt and rains, suggesting that the source of nitrate may actually be surrounding groundwater.^^13^^

The SBX2 1 report focused on nitrate pollution in California’s Tulare Lake Basin and Salinas Valley, a region that comprises four of the five most agriculturally productive counties in the United States, and that supplies hundreds of different foods to the entire continent throughout the year. The soil there has been cultivated intensively since World War II, and nitrate concentrations in the groundwater in many locations regularly exceeded the state’s maximum contaminant level of 45 mg/L between 2006 and 2010. Some 250,000 people using that groundwater obtain it from treatment systems that are not equipped to deal with this kind of contamination.^^1^^

## Solutions: Simple but Not Easy

SBX2 1 author Harter cautions that there is no easy fix for nitrate pollution in groundwater, which will likely continue to worsen absent a shift in agricultural policy and practice. The vast majority of this nitrate pollution is the result of long-term fertilizer application, and the most recently applied material will continue to make its way into aquifers for decades to come. Nor are there good technical solutions for removing this contamination *in situ*, which would be inordinately expensive.

However, SBX2 1 features a range of promising solutions to the challenge posed by reactive nitrogen, all of which the authors say could be implemented without the need for further scientific research or technological development. What *is* required is policy direction, including the enforcement of existing regulations or the introduction of new legislation to improve public oversight of water management.

One cost-effective approach cited by SBX2 1 is the use of nitrogen-rich groundwater to irrigate and fertilize crops in a more efficient way. Other practical approaches would deal with reactive nitrogen closer to its source, so that it does not have to be managed in soil or water. For instance, constructed wetlands or bioreactors installed on the periphery of agricultural settings could capture and treat nitrate-rich runoff before it enters the wider environment.

Unfortunately, this kind of infrastructure represents a significant financial outlay, one that would also add to the workload of agricultural operators. The cash-strapped California government could not easily subsidize these facilities, nor would farming communities readily take on the burden, according to the authors. A simpler strategy might be a tax on the use of fertilizer, which would provide a direct incentive to apply less of this material on the land and so reduce the amount of nitrogen going into the soil. A similar tax could be imposed on the flow of nitrogen, as measured in farm runoff and other wastewater streams. But depending on how such a tax were applied, modeling suggests farm output and revenue could decrease.^^1^^

The report points to the crucial role of monitoring and assessment by bodies such as regional and state water boards. But throughout their study, the SBX2 1 authors wrote that “we often faced insurmountable difficulties in gaining access to data already collected on groundwater and groundwater contamination by numerous local, state, and federal agencies. Inconsistencies in record keeping, labeling, and naming of well records make it difficult to combine [data] on the same well that exist in different databases or that were collected by different agencies. A statewide effort is needed to integrate diverse water-related data collection activities of various state and local agencies with a wide range of jurisdictions.”^^1^^ The authors suggest these activities could be enhanced with the support of fertilizer excise and water-use fees.

Among those who would be on the front line of such oversight is Celeste Cantu, general manager of the Santa Ana Watershed Project Authority. As part of the panel discussion following the release of SBX2 1, she emphasized the cooperative nature that any solution will have to take.

“There is no silver bullet, but we have a lot of silver buckshot,” she said, noting that any workable solution would have to be multi-faceted, spreading a variety of costs and benefits among a variety of partners. “It’s only going to be crafted on a region-by-region basis, each time as unique as the soils are, unique as the circumstances are,” she said.

As for sites where drinking water quality has already been compromised and will remain so for some time to come, the authors argue that point-of-use water treatment represents the most feasible option. In the short term, individual households could be helped with the purchase of the necessary equipment for removing nitrate contamination. Eventually, this assistance could extend to smaller municipalities, helping them upgrade their water-treatment operations or integrate those operations with larger regional plants already capable of eliminating nitrates.

## Pinning Down the Fate of Reactive Nitrogen

But such measures are just the beginning of the work that lies ahead, says Tom Tomich, a professor of community development, environmental science, and policy at UC Davis. He is the principal investigator on a separate but related project, the California Nitrogen Assessment,^^14^^ which has been running for about three years with a broad range of participation, from local activist groups to key agricultural producers. That undertaking was inspired by another piece of California legislation, the state’s global warming bill AB 32,^^15^^ which brought scientists and regulators together to consider emissions of N_2_O, a highly potent greenhouse gas.

Nitrogen is now understood to help regulate the carbon cycle and exert both cooling and warming effects on the climate.^^5^^ For example, nitrogen compounds can increase carbon sequestration—a cooling effect—by stimulating tree growth and slowing the decay of organic material in some soils. They can also contribute to particulate pollution, which imparts a short-term cooling effect by modulating the sun’s radiation. On the other hand, long-lived N_2_O has about 300 times the warming potential of carbon dioxide.

Although atmospheric data indicate that N_2_O represents only a minor proportion of all greenhouse gases,^^16^^ some more sobering insights emerged from the California Nitrogen Assessment. For instance, only about half of all the nitrogen applied to soils as fertilizer actually becomes part of the subsequent crop; the rest is lost to the environment.^^17^^ “For every nitrogen atom coming into the state,” says Tomich, “one quarter of those ended up in groundwater as nitrate.”

According to Alan Townsend, a coauthor of the *Issues in Ecology* report who currently serves as director of the National Science Foundation Division of Environmental Biology, the larger challenge facing nitrogen pollution research efforts is that they depend on indirect tracking of nitrogen flow. In a 2008 *Science* article, Townsend and colleagues maintained that a great deal of uncertainty dogs any attempts to pin down the fate of reactive nitrogen.^^7^^ Some two-thirds of the total may be accumulating in soils, vegetation, and groundwater, they suggest. From there it could be denitrified into simple N_2_ and emitted into the atmosphere, but the ultimate fate of that accumulating nitrogen remains unclear.^^18^^

“The faster reactive nitrogen is denitrified, the faster you’ve taken one of those reactive nitrogen molecules out of its ‘crime spree’ and put it back to where it’s not hurting us,” says Townsend. “But it is very hard to measure that process at any scale above a really small one, such as a lab or a controlled field condition.”

In geological terms, Townsend and colleague Stephen Porder pointed out in a new report, humanity’s major disruption of the natural nitrogen cycle has lasted no longer than the blink of an eye. We therefore have only a short window of observation upon which to predict the ultimate environmental outcome of this disruption, whose “legacy will be with us for generations.”^^19^^ Meanwhile, the global manufacture of reactive nitrogen accelerates and could continue to do so, depending on whether nations alter their agricultural strategies.^^20^^

Nevertheless, Townsend regards the dramatic success of the Clean Air Act^^21^^ in reducing reactive nitrogen in the atmosphere—which the EPA estimates prevented nearly 165,000 premature U.S. deaths in 2010 alone^^22^^—as a testament to what could be accomplished on the ground in relatively short order. “You see that it can work without some sort of gigantic societal or economic upheaval,” he says.

In this context, he regards initiatives like SBX2 1 and the California Nitrogen Assessment as models for how to nurture public and private support for policies that have already demonstrated their value. He says, “Not just outlining the problem and getting the data, but getting in deep with the stakeholders and talking with all of them, and seeking common ground and solutions—this is a way forward.”
